# Spin reorientation and controllable magnetic anisotropy in two-dimensional MnSe2/As van der Waals heterostructures

**DOI:** 10.1016/j.isci.2025.113830

**Published:** 2025-10-23

**Authors:** Wei Chen, Yunpeng Lan, Jujian Liao, Youneng Guo

**Affiliations:** 1 School of Electronic Information and Electrical Engineering, Changsha University, Changsha 410022, China; 2 Shenzhen State Microelectronics CO., LTD, Shenzhen 518063, China; 3 School of Physics and Chemistry, Hunan First Normal University, Changsha 410205, China

**Keywords:** Physics, Nanoscience

## Abstract

First-principles calculations demonstrate that interfacial coupling in MnSe_2_/As van der Waals heterostructures induces spin reorientation, switching the easy-magnetization axis of the MnSe_2_ monolayer from in-plane to out-of-plane. Interlayer charge transfer generates a built-in electric field directed from the As layer toward MnSe_2_, which drives this spin reorientation. The magnetocrystalline anisotropy energy (MAE) is found to be broadly tunable via external electric fields, interlayer distance variation, and thickness of the As layer. Crucially, perpendicular magnetic anisotropy (PMA) enhancement is achieved via applied electric fields and reduced interlayer spacing. Atomic-resolved MAE and orbital-resolved MAE analysis combined with density of states calculations reveal that the PMA enhancement primarily originates from modified electronic states of Se-*p* orbitals. This work establishes a viable strategy for tailoring 2D magnetic properties and may be helpful for preparing 2D spintronic devices.

## Highlights

• The MnSe_2_/As heterostructure exhibits high-*T*_C_ ferromagnetism and strong PMA
• Built-in electric field induces spin reorientation, shifting anisotropy from IMA to PMA
• MAE is tunable via electric field, interlayer distance, and As-layer thickness
• PMA enhancement primarily originates from modified electronic states of Se-p orbitals

## Graphical abstract

**Figure:**
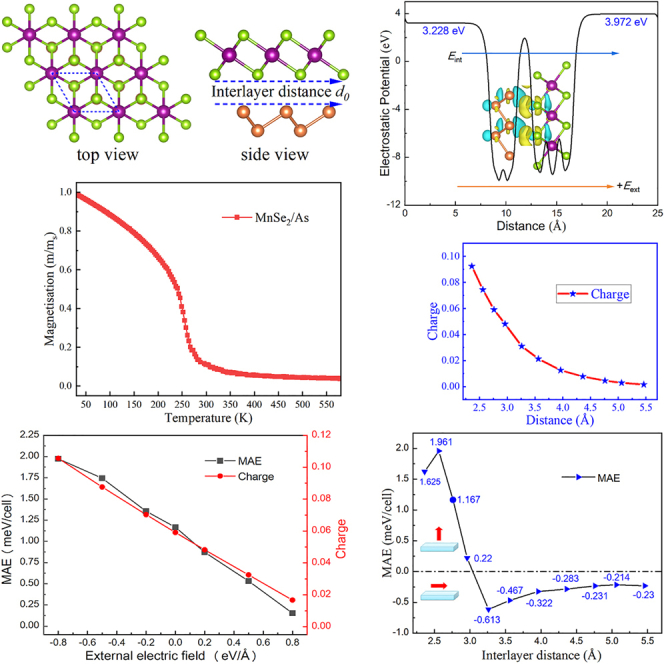

## Introduction

Intrinsic long-range magnetic order has been experimentally realized in two-dimensional (2D) van der Waals (vdW) magnets, such as atomically thin Cr(Cl/Br/I)_3_, Cr_2_Ge_2_Te_6_, MnBi_2_Te_4_, Fe_3_GeTe_2_, and so on.^1^^,^^2^^,^^3^^,^^4^^,^^5^^,^^6^^,^^7^^,^^8^^,^^9^^,^^10^ This breakthrough holds significant promise for next-generation spintronics and ultrahigh-density magnetic storage.^2^^,^^11^^,^^12^^,^^13^^,^^14^^,^^15^ However, the stability of long-range magnetic order in these 2D systems at finite temperatures is constrained by the Mermin-Wagner theorem.^13^^,^^16^ Crucially, this necessitates strong uniaxial magnetic anisotropy to suppress thermal fluctuations and stabilize 2D magnetism at finite temperatures. Moreover, compared to in-plane magnetic anisotropy (IMA), perpendicular magnetic anisotropy (PMA) holds particular technological promise, offering superior thermal stability, reduced critical switching currents, and better scalability for device fabrication.^17^^,^^18^^,^^19^^,^^20^^,^^21^^,^^22^^,^^23^

Recent breakthroughs in synthesis now enable the fabrication of free-standing MnSe_2_ monolayers (MLs) on GaSe_2_/SnSe_2_ substrates.^23^ These MLs exhibit the highly sought-after characteristic of room-temperature ferromagnetic ordering, although with weak PMA. First-principles calculations indicate that the pristine MnSe_2_ ML possesses an intrinsic in-plane easy axis. Furthermore, recent theoretical studies suggest that its magnetic properties can be modulated via methods such as charge doping, biaxial strain, and so on.^21^^,^^24^^,^^25^^,^^26^^,^^27^ Nevertheless, the development of 2D ferromagnets that simultaneously achieve high magnetic ordering temperatures and strong PMA remains a significant challenge.

A promising strategy to engineer magnetic properties in 2D materials involves constructing vdW heterostructures. Recent studies highlight the efficacy of interfacing 2D magnetic layers with 2D Group VA semiconductors (i.e., As, Sb, and Bi).^28^^,^^29^^,^^30^ For instance, integrating Bi with CrI_3_ MLs has enabled quantum anomalous Hall effect, spin reorientation, and enhanced Curie temperature (*T*_C_).^28^^,^^29^ Compared to the pure MnTe_2_ ML, the Sb/MnTe_2_ heterostructures demonstrate significantly improved PMA and *T*_C_.^30^ Notably, the magnetic properties of vdW structures exhibit exquisite sensitivity to the interlayer spacing and electric field.^15^^,^^20^^,^^29^^,^^31^^,^^32^^,^^33^ Experimentally, it is found that for bilayer CrI_3_, the hydrostatic pressure modulates magnetic order,^31^ and voltage control switching between antiferromagnetic and ferromagnetic states.^15^ Theoretical research indicates that giant PMA is obtained in graphene/NiI_2_ and Bi/CrI_3_ heterostructures by tuning interlayer distance,^20^^,^^29^ and vertical electric fields drastically alter anisotropy directions in Mn_2_CFCl/MoSSe heterostructure.^32^ These findings collectively establish interlayer distance and electric field as potent approaches for tuning magnetic anisotropy in vdW heterostructures.

In this paper, we have leveraged the negligible lattice mismatch between MnSe_2_ and As MLs to construct MnSe_2_/As vdW heterostructures. Although Sb or Bi MLs possess stronger spin-orbit coupling (SOC) and could potentially enhance magnetocrystalline anisotropy, their heterostructures with MnSe_2_ face challenges. These include substantial lattice mismatch and poor environmental stability, with Bi being particularly prone to oxidation. In contrast, the MnSe_2_/As system employs a distinct mechanism based on interfacial coupling effect that enables strong PMA without relying on heavy elements. Using first-principles calculations, we demonstrate that interfacial coupling reorients the easy-magnetization axis of MnSe_2_ from in-plane to out-of-plane. Furthermore, the magnetocrystalline anisotropy energy (MAE) is tunable via external electric fields, interlayer distance, and thickness of As layers. Notably, PMA enhancement in MnSe_2_ can be achieved through external electric field application and reduced interlayer spacing. Atomic-resolved MAE, orbital-resolved MAE, and density of states (DOS) analyses reveal that the enhancement of magnetic anisotropy in MnSe_2_ primarily originates from the electronic state variations of interfacial Se atoms.

### Computational methods

First-principles calculations were performed using the Vienna ab initio simulation package based on density functional theory (DFT).^34^ The projector augmented wave method^35^ described ion-electron interactions, and the Perdew-Burke-Ernzerhof functional within the generalized gradient approximation (GGA) handled exchange-correlation energy.^36^ An on-site Coulomb interaction (U = 3.9 eV)^21^^,^^30^^,^^37^^,^^38^^,^^39^ was applied to Mn-d orbitals to account for electron correlations. A 500 eV plane-wave energy cutoff was set, with convergence criteria of less than 10^−6^ eV for the energy difference between electronic steps and below 10^−4^ eV Å^−1^ for the force on each atom. Γ-centered Monkhorst-Pack grids of 18 × 18 × 1 sampled the first Brillouin zone for the MnSe_2_/As heterostructures. For MAE calculations, non-collinear calculations are considered SOC. The energy cutoff was increased to 600 eV with a 10^−7^ eV convergence criterion. All calculations included D3 VdW correction^40^ for interlayer interactions and a 20 Å vacuum layer along the *z*-direction to avoid vertical interactions.

The MAE is calculated using the GGA + U + SOC method, defined as the energy difference between the out-of-plane magnetization orientation (*E*_⊥_, aligned with the [001] axis) and the in-plane orientation (*E*_∥_, aligned with the [100] axis): MAE = *E*_∥_-*E*_⊥_. Self-consistent calculations were performed to determine the total energies for both magnetization directions.^29^^,^^41^ The sign of MAE dictates the easy magnetization axis: a negative MAE indicates preferential in-plane magnetization, while a positive MAE corresponds to an out-of-plane easy axis. Assuming SOC is treated perturbatively, MAE can be expressed as^42^^,^^43^

(Equation 1) $$\text{MAE}=\xi^{2}\sum_{\sigma ,\sigma^{\prime }}\left(2\delta_{\sigma {,\sigma }^{\prime }}-1\right)\sum_{o^{\sigma }{,u}^{\sigma^{\prime }}}\frac{{\left|\left\langle o^{\sigma }\left|L_{z}\right|u^{\sigma^{\prime }}\right\rangle \right|}^{2}-{\left|\left\langle o^{\sigma }\left|L_{x}\right|u^{\sigma^{\prime }}\right\rangle \right|}^{2}}{E_{u}^{\sigma^{\prime }}-E_{o}^{\sigma }}\;$$

where *ξ* is the SOC constant, *o*^*σ*^ and $u^{\sigma^{\prime }}$ denote occupied and unoccupied states with spins σ and $\sigma^{\prime }$ (majority/minority), respectively, and $E_{o}^{\sigma }$, $E_{u}^{\sigma^{\prime }}$ are their corresponding energies. The factor $\left(2\delta_{\sigma \sigma^{\prime }}-1\right)$ equals +1 for same-spin transitions (σ = $\sigma^{\prime }$) and −1 for opposite-spin transitions (σ ≠ $\sigma^{\prime }$). For same-spin transitions, the orbital coupling through *L*_*z*_(*L*_*x*_) yields a positive (negative) contribution to MAE. The second-order perturbation expression (Equation 1) for MAE explicitly demonstrates that heavy elements with large SOC constants *ξ* are prime candidates for achieving giant MAE.

## Results and discussion

### Crystal structure, stability, and magnetic properties

**Figure 1:**
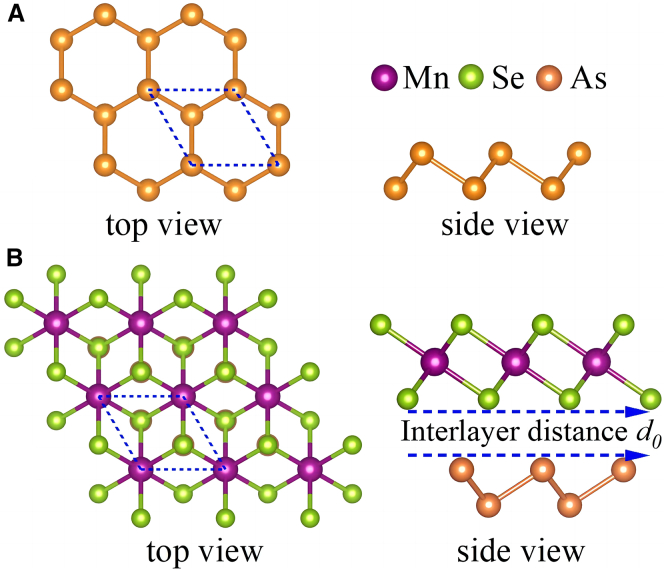
Top and side views of the crystal structures (A) *β*-phase As monolayer. (B) MnSe_2_/As heterostructure.

Figures 1A and 1B illustrate the crystal structures of As ML and the vertically stacked MnSe_2_/As bilayer vdW heterostructure, constructed by integrating the MnSe_2_ and As MLs. Prior to heterostructure assembly, individual lattice structure optimizations are performed for each constituent. First-principles calculations reveal that ML As exhibits a graphene-like hexagonal *β-*phase crystal structure with the $P\bar{3}m1$ space group, as shown in Figure 1A. MnSe_2_ ML, also belonging to $P\bar{3}m1$ space group, is observed to exhibit an octahedral T-phase. In the top-view projection, each Mn atom is coordinated with six Se atoms, forming a perfect octahedral configuration. The side-view perspective highlights the sandwich-like architecture, where the Mn atomic layer is positioned between two Se atomic layers. The optimized lattice constants *a*_0_ of As and MnSe_2_ MLs are 3.630 Å and 3.653 Å, respectively, which are in excellent agreement with previously reported results.^24^^,^^30^^,^^39^ These structural parameters validate the rationality of constructing the vdW heterostructure via vertical stacking, as the minimal lattice mismatch facilitates stable interfacial coupling.

To mitigate artificial internal strain from lattice mismatch, the MnSe_2_/As heterostructure employs a unit cell of As ML matched to the primitive cell of MnSe_2_ ML. As shown in Figure 1B, top-view alignment reveals near-complete spatial overlap between As and Se atoms. The lattice misfit (*ε*) is calculated using

(Equation 2) $$\varepsilon =\frac{\left|\alpha_{1}-\alpha_{2}\right|}{\left(\alpha_{1}+\alpha_{2}\right)/2}\times 100\%$$

where α_1_ and α_2_ denote the relaxed lattice parameters of the two MLs. In the optimized heterostructure, the lattice mismatch is as low as ∼0.64%, indicating very small strain and good growth compatibility with an As substrate. This value is much lower than the ∼5% typically required for epitaxial growth of 2D heterostructures,^44^ and even lower than that of other systems like ReSe_2_/MoSe_2_ (∼2.06%) where epitaxial growth has been achieved.^45^ The heterostructure’s lattice constant (*a*_0_ = 3.648 Å) closely matches that of MnSe_2_ ML, while the interlayer distance (*d*_0_ = 2.761 Å) exceeds the sum of interfacial atomic radii, confirming vdW interactions. Four vertical stacking configurations (see Figure S1 of the supplemental information) were evaluated, showing minimal energy differences that imply comparable stability. The lowest-energy configuration was selected for subsequent analyses.

The binding energy *E*_*bind*_ of the MnSe_2_/As heterostructure is defined as

(Equation 3) $$E_{bind}=\frac{{\;E}_{h}-\left({E_{{MnSe}_{2}}+E}_{As}\right)}{N}$$

where *E*_*h*_, $E_{{MnSe}_{2}}$, and *E*_*As*_represent the total energies of the MnSe_2_/As heterostructure, MnSe_2_ ML, and As ML, respectively. N (*N* = 5) denotes the total number of atoms in the heterostructure unit cell. A negative *E*_*bind*_ indicates stable heterostructure formation, as energy is released during assembly. For the MnSe_2_/As heterostructure, the calculated *E*_*bind*_is −175 meV, confirming that MnSe_2_ ML maintains structural stability when coupled with As ML. This exothermic binding process signifies strong interfacial compatibility, consistent with the low lattice mismatch (*δ* = 0.64%) and vdW interaction characteristics.

**Figure 2:**
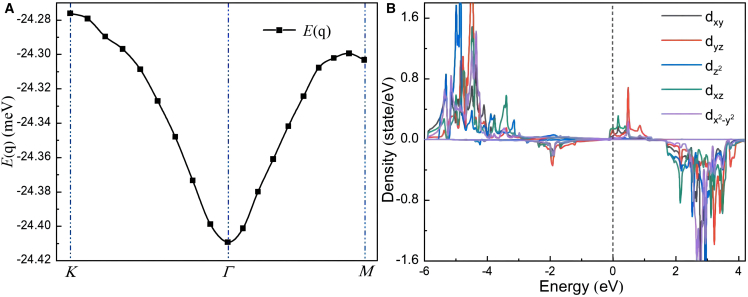
Spin-helical energy dispersion relation and Mn-*d* orbital PDOS of MnSe_2_/As heterostructure (A) Spin-helical energy dispersion relation *E*_*J*_(*q*). (B) The PDOS of the Mn-d orbitals. In (A), discrete points represent the energy dispersion *E*_*J*_(*q*) as a function of the spin-helical wave vector q obtained from program calculations, and lines are fitted ones.

According to the generalized Bloch theorem, the spin helical state can be described by the generalized translation operator combining spatial translation and spin rotation, where the spin helical wave vector *q* characterizes the periodicity and direction of the non-collinear magnetic structure.^46^ For a hexagonal lattice, if the minimum of the spin helical energy dispersion *E*(*q*) appears at the *Γ* point (*q* = 0), it corresponds to the ferromagnetic state with all spins parallelly aligned. As shown in Figure 2A, the *E*(*q*) of the MnSe_2_/As heterojunction is expanded along the high-symmetry path *K-Γ-M* in the 2D Brillouin zone, and the energy minimum is located at the *Γ* point, indicating that the ground state of this system is ferromagnetic order, tending to have spins parallelly aligned.

Using the anisotropic Heisenberg model, we computed the exchange interactions in both the MnSe_2_ ML and the MnSe_2_/As heterostructure, with detailed computational methods for the exchange constants and *T*_C_ provided in Data S1 of the supplemental information. As shown in Figure S2 of the supplemental information, the isotropic exchange interaction (*J*) in the heterostructure nearly equals that of the MnSe_2_ ML. The symmetric anisotropic exchange (*Jani*) exhibits a slight reduction. Notably, a significant Dzyaloshinskii-Moriya interaction (*D*) is introduced in the heterostructure. Based on these magnetic interaction parameters, we simulated the temperature dependence of the average magnetic moment, and the results are presented in Figure S3. The estimated *T*_C_ of the MnSe_2_/As heterostructure is approximately 265 K, which is slightly lower than that of the pristine MnSe_2_ ML (∼300 K). These results indicate that the MnSe_2_/As heterostructure still maintains high-*T*_C_ ferromagnetism.

The total magnetic moment (*S*_*tot*_) of the MnSe_2_/As heterostructure equals that of MnSe_2_ ML, at 3.0 *μ*_B_. Within the heterostructure, the local magnetic moment on the Mn atom (*S*_Mn_) is 3.98 *μ*_B_, while the average moments on Se and As atoms are −0.35 and −0.03 *μ*_B_, respectively. The fact that *S*_Mn_ exceeds *S*_*tot*_ reflects the local character of the Mn moment within its Wigner-Seitz radius. The majority of the magnetic moment resides on the Mn atom, with the adjacent Se atoms exhibiting small negative moments, indicating antiferromagnetic coupling to Mn. This leads to a net total moment of 3.0 *μ*_B_ per unit cell. As shown in Figure 2B, the partial density of states (PDOS) of Mn-*d* orbitals confirms that the spin-polarized bands near the Fermi level (*E*_*F*_) originate primarily from localized Mn-d states, further supporting the dominant contribution of Mn to the total magnetism. In the MnSe_2_/As heterostructure, which belong to the D_3*d*_ point group, the crystal field splits the 5-fold degenerate Mn-d orbitals into three sets: a single degenerate state (*a*_1g_), and two 2-fold degenerate states (*e*_g_ and *t*_2g_). The PDOS of Mn atoms shows strong exchange splitting of Mn-*d* orbitals. The five Mn-*d* orbitals with different orientations become delocalized due to orbital overlap and hybridization. Following Hund’s rule and the Pauli exclusion principle,^47^^,^^48^ spin-up (majority-spin) orbitals are nearly fully occupied by five unpaired Mn^5+^ *d* electrons, while spin-down (minority-spin) states are partially occupied. This results in *S*_*Mn*_ being slightly smaller than the magnetic moment of an isolated Mn atom (calculated as 5.0 *μ*_B_).

### Spin reorientation and origin of magnetic anisotropy

Calculations demonstrate that coupling between MnSe_2_ with As MLs induces reorientation of the easy-magnetization axis. For pure MnSe_2_ ML, the easy-magnetization axis lies in the in-plane direction, accompanied by an IMA of −0.39 meV/cell. In contrast, the MnSe_2_/As heterostructure exhibits an out-of-plane easy-magnetization axis with a significantly enhanced PMA of 1.18 meV/cell. A critical observation is that the MAE in both systems is predominantly governed by Se atoms. In the pure MnSe_2_ ML, Se atoms contribute −0.12 meV/cell to the MAE, whereas Mn atoms make negligible contributions. For the MnSe_2_/As heterostructure, Se atoms contribute 1.05 meV/cell to the MAE, while the contributions from As and Mn atoms are negligible. In the single-ion model, MAE arises from the combined SOC effects of magnetic atoms and the crystal field splitting of their orbital states. Notably, prior research has indicated that the SOC of heavy elements also plays a crucial role in determining MAE, as observed in materials such as MnTe_2_, CrI_3_, InCrTe_3_, and so on.^12^^,^^20^^,^^29^^,^^37^ Calculating the MAE of the pure MnSe_2_ ML as a function of the SOC strength of specific elements demonstrates that the MAE originates primarily from the SOC of nonmagnetic Se atoms.^21^ This finding suggests that the single-ion model cannot fully account for the magnetic anisotropy in MnSe_2_ ML, a conclusion that also applies to the MnSe_2_/As heterostructure.

**Figure 3:**
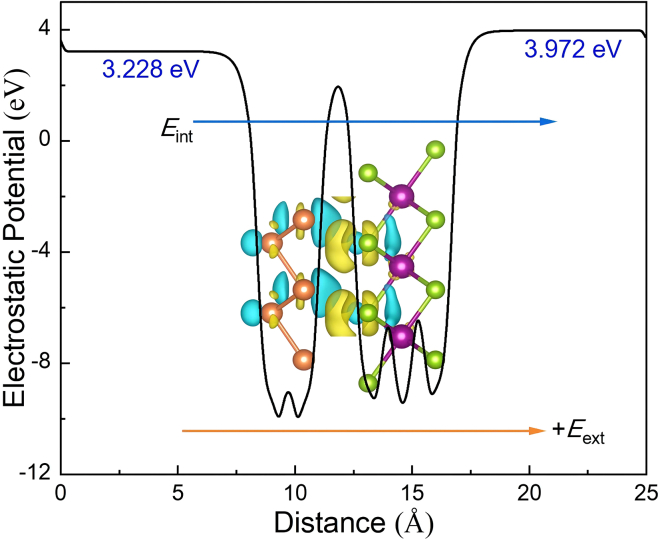
Electrostatic potential distribution along the *c*-axis and differential charge density of MnSe_2_/As heterostructure Sky blue and yellow regions represent electron loss and accumulation, respectively. The contour value is 0.00033 e/bohr3.

The MnSe_2_/As heterostructure exhibits minimal lattice deviation relative to the pure MnSe_2_ ML, indicating that strain effects are unlikely to drive the magnetization reorientation and PMA enhancement. To further validate this, additional MAE calculations were performed for a strained MnSe_2_ ML using the heterostructure’s lattice parameters. The resulting MAE value (calculated as −0.199 meV/cell) shows negligible deviation from that of the pure MnSe_2_ ML, providing direct evidence that lattice strain does not govern the observed MAE transition. Instead, the spin reorientation and enhanced PMA in the MnSe_2_/As heterostructure are primarily driven by interfacial charge transfer, as confirmed by differential charge density analyses (see Figure 3) and Bader charge calculations. These reveal a charge transfer of ∼0.059 electrons per unit cell from As (the donor) to MnSe_2_ (the acceptor), with charge accumulation at the As-Se interface. This charge redistribution induces a built-in electric field (*E*_int_ = 0.21 eV/Å) oriented from the As layer to MnSe_2_, as evidenced by the electrostatic potential gradient in Figure 3. The asymmetric geometry of the heterostructure stabilizes the built-in electric field, which modulates the crystal field around Se atoms and drives the magnetization reorientation.

**Figure 4:**
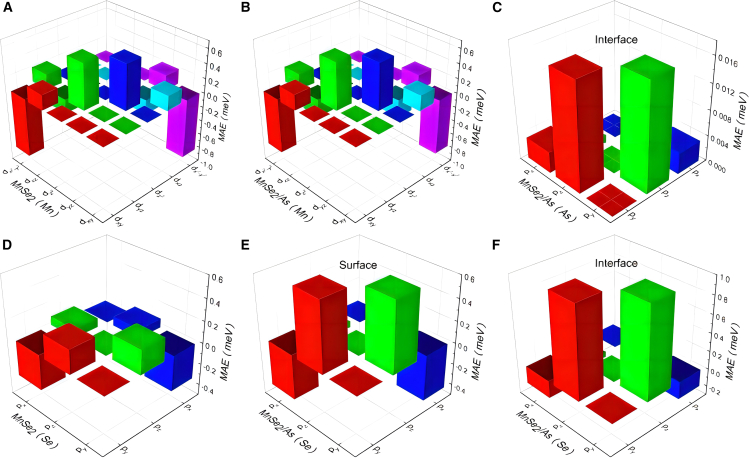
Orbital-projected contributions to MAE from Mn, Se, and As atoms (A and B) Mn atoms’ contributions in (A) pure MnSe_2_ monolayer and (B) MnSe_2_/As heterostructure. (C) Interfacial As atom’s contribution in heterostructure. (D) Se atom’s contribution in pure monolayer. (E and F) (E) Interfacial and (F) surface Se atoms’ contributions in heterostructure, respectively.

Figures 4A–4F present the orbital-projected contributions to MAE from Mn, Se, and As atoms in pure MnSe_2_ ML and MnSe_2_/As heterostructure. When MnSe_2_ ML is coupled with As ML, the orbital-projected MAE contributions from Mn atoms exhibit negligible changes, as shown in Figures 4A and 4B. For both the MnSe_2_ ML and the MnSe_2_/As heterostructure, the in-plane contribution (negative) to the MAE mainly arises from SOC through the Mn atom’s $\left(d_{x^{2}-y^{2}},d_{xy}\right)$ orbitals, while the coupling between the (*d*_*yz*_, *d*_*xz*_) orbitals contributes most out-of-plane value (positive). Significantly, the negative and positive MAE contributions from Mn-*d* orbitals nearly cancel each other out. The orbital-projected contributions to MAE from As atoms are negligible, as shown in Figure 4C. The Se atoms dominate the orbital-projected MAE in both the MnSe_2_ ML and the MnSe_2_/As heterostructure. In MnSe_2_ ML, the in-plane contribution to the MAE arises from SOC through the Se atom’s (*p*_*x*_, *p*_*y*_) orbitals, which outweighs the out-of-plane contributions from SOC between the (*p*_*y*_, *p*_*z*_) and (*p*_*x*_, *p*_*z*_) orbitals, resulting in a net negative MAE, as shown in Figure 4D. Notably, MnSe_2_ ML grown on GaSe/SnSe_2_ substrates displays weak PMA, a phenomenon potentially attributable to strain effects induced by the substrates.^23^ Upon forming the heterostructure with As ML, the Se atoms’ contributions to MAE exhibit a significant shift. In the MnSe_2_/As heterostructure, there are two distinct Se atom layers, namely the interfacial and surface Se atoms, which contribute differently to the MAE. As shown in Figure 4E, for the interfacial Se atoms, the positive contribution from SOC through Se atom’s (*p*_*z*_, *p*_*y*_) orbitals significantly increases, while the negative contribution from SOC through the (*p*_*x*_, *p*_*y*_) markedly decreases. Similar changes occur in the surface Se atoms (see Figure 4F), although their MAE contributions are much smaller compared to those of the interfacial Se atoms. Overall, these alterations in the orbital-projected contributions from Se atoms drive the reorientation of the easy magnetization direction from in-plane to out-of-plane.

**Figure 5:**
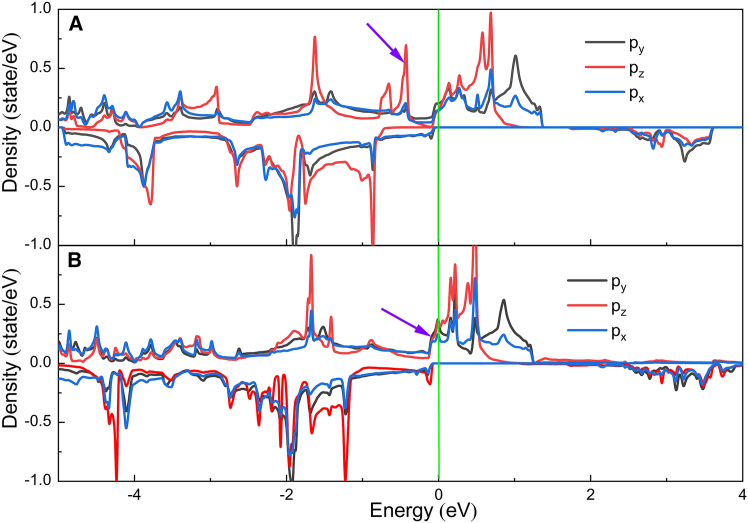
PDOS of Se-*p* orbitals for two systems (A) Pure MnSe_2_ monolayer. (B) MnSe_2_/As heterostructure.

To further unravel the spin reorientation phenomenon in the MnSe_2_/As heterostructure, we examine how interlayer coupling affects the electronic configurations. Given that Mn atoms, and the As layer have little effect on MAE, we focus on the impact of electron occupancy in Se-*p* orbitals on MAE, using second-order perturbation theory as the theoretical foundation. Specifically, comparative analysis of PDOS for Se atoms in the MnSe_2_ ML and the MnSe_2_/As heterostructure enables qualitative insight into the fundamental origins of altered MAE contributions. As illustrated in Figure 5A, in MnSe_2_ ML, the couplings of the $\left(p_{x\left(y\right)}^{u^{+}},p_{y\left(x\right)}^{o^{-}}\right)$ and $\left(p_{z}^{u^{+}},p_{x\left(y\right)}^{o^{-}}\right)$ states dominate the MAE contributions as described by the second-order perturbation theory. As indicated in Table S1 of the supplemental information, the coupling of $\left(p_{x\left(y\right)}^{u^{+}},p_{y\left(x\right)}^{o^{-}}\right)$ states exhibits a value of −1, contributing to IMA, while the coupling of $\left(p_{z}^{u^{+}},p_{x\left(y\right)}^{o^{-}}\right)$ states has a value of +1, favoring PMA. This is consistent with the trends depicted in Figure 4D. The interplay between these two interactions leads to the easy-magnetization direction of Se atoms aligning parallel to the *x* axis. In the MnSe_2_/As heterostructure, a strong built-in electric field induces a change in the originally $p_{z}^{u^{+}}$ states to $p_{z}^{u^{-}}$ states near the Fermi level (*E*_F_), as denoted by the arrows in Figure 5B. Concurrently, the $p_{x\left(y\right)}^{o^{+}}$ states near the *E*_F_ experience a slight increase. This shift brings about a comprehensive modification of the interactions among electronic states. Currently, the key interactions around the *E*_F_ occur between $\left(p_{x\left(y\right)}^{u^{+}},p_{x\left(y\right)}^{o^{+}}\right)$ and $\left(p_{x\left(y\right)}^{u^{+}},p_{z}^{o^{-}}\right)$ states. Referring to Table S1, both of these interactions have a value of +1, consequently resulting in a significant PMA. These results respectively match the trends illustrated in Figures 4E and 4F. This theoretical analysis is consistent with the outcomes of the direct DFT + U + SOC calculations.

The electronic band structure plays a critical role in the spin reorientation of the MnSe_2_/As heterostructure. The projected band structures, shown in Figure S4 of the supplemental information, indicate that the states near *E*_F_ originate from As, Mn, and Se atoms. Strong hybridization between Mn and Se orbitals is responsible for the anisotropic exchange interaction.^49^ Due to exchange interaction between Mn and Se, the spin orientation of Mn atoms aligns parallel to that of Se, along the *z* axis, which corresponds to PMA. Moreover, notable hybridization between As and Se orbital states occurs near *E*_F_. This interfacial hybridization facilitates charge transfer, thereby altering the occupation of Se-p orbitals. Such modification directly modulates the MAE and promotes the transition from in-plane to out-of-plane spin orientation.

### Tuning MAE with external electric fields

**Figure 6:**
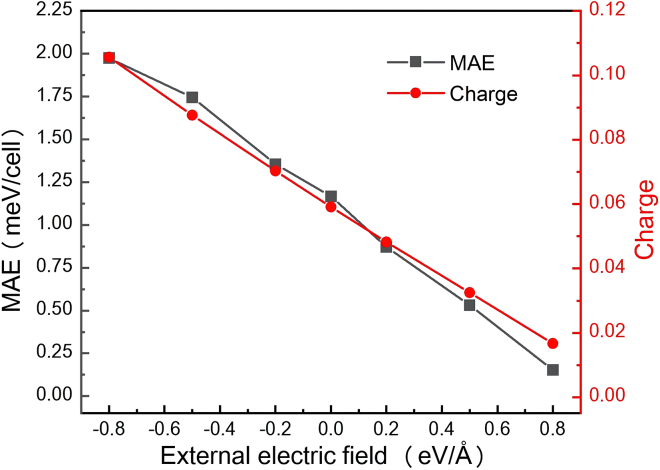
External electric field’s influence on MAEs and charge transfer amount of MnSe_2_/As heterostructure Negative values denote that the *E*_ext_ is directed from MnSe_2_ to As, while positive values indicate the opposite direction.

In the MnSe_2_/As heterostructure, MAE can be flexibly engineered by magnitude of the external electric field (*E*_ext_). Figure 6 illustrates the *E*_ext_-dependent evolution of MAE and the charge transfer in the heterostructure. Specifically, the *E*_ext_ directly influences the amount of charge transfer from As to MnSe_2_. Larger charge transfer correlates with stronger PMA. Under a strong negative *E*_ext_ of −0.8 eV/Å, the amount of charge transfer reaches ∼0.106 electrons, corresponding to a maximum MAE of 1.98 meV/cell. Conversely, a positive *E*_ext_ reduces the charge transfer to only ∼0.016 electrons at 0.8 eV/Å, weakening interfacial interactions and decreasing MAE to 0.15 meV/cell. These results demonstrate a strong correlation among the charge transfer, the MAE modulation, and the external electric field. This mechanism provides a crucial strategy for tailoring magnetic properties in vdW heterostructures for spin-based device applications.

**Figure 7:**
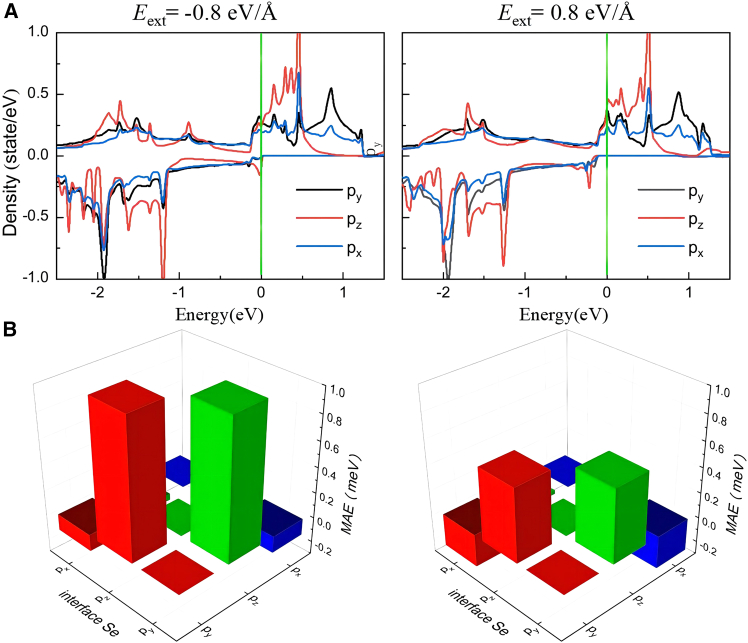
PDOS and orbital-projected MAE contribution of Se atoms in MnSe_2_/As heterostructure under different external electric fields (A) PDOS of Se atoms. (B) Contribution to MAE from interfacial Se atoms.

To investigate the PMA modulation mechanism, we have enumerated the PDOS and the orbital-projected MAE contributions from interfacial Se atoms in the MnSe_2_/As heterostructure under the external electric fields of −0.8 and 0.8 eV/Å. Our calculations show the modulation of PMA is predominantly attributed to the orbital contribution variations of interfacial Se atoms, while the SOC contributions exhibit negligible changes for surface Se atoms. As indicated in Figures 7A and 5B, as the *E*_ext_ varies from 0.8 eV/Å to 0 eV/Å to −0.8 eV/Å, the $p_{z}^{o^{-}}$ states near the *E*_F_ continuously shift toward higher energies (rightward). Concurrently, the $p_{x\left(y\right)}^{o^{-}}$ states near the *E*_F_ show a slight reduction. The former and the latter exhibit values of +1 and −1, respectively (as shown in Table S1). Consequently, the PMA gradually improves. The orbital-projected MAE contributions demonstrate a direct response to PDOS variations, as shown in Figures 7B and 4E. When the *E*_ext_ switches from negative to positive, the positive SOC contribution to MAE through the (*p*_*z*_, *p*_*y*_) orbitals decreases significantly, while the negative SOC contribution between the (*p*_*x*_, *p*_*y*_) orbitals shows a marginal increase.

### Tuning MAE via interlayer distances

**Figure 8:**
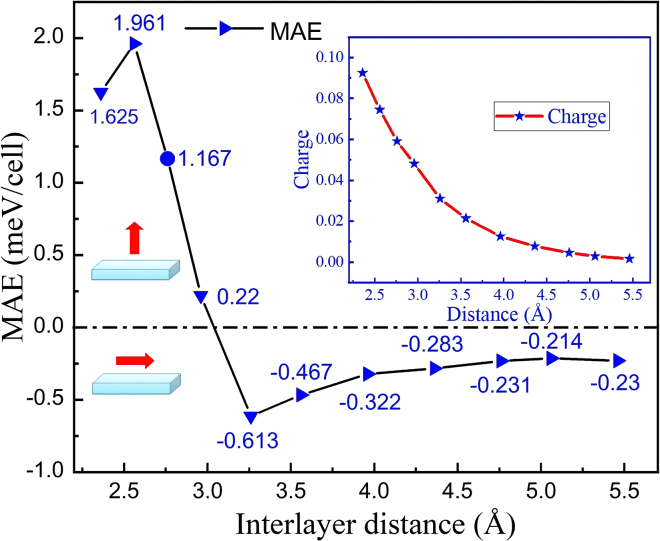
Interlayer distances influence on MAEs and charge transfer amount of MnSe_2_/As heterostructure

The interlayer coupling effect significantly governs the electronic properties of heterostructures. Hence, we have delved deeper into the MAE of the MnSe_2_/As heterostructure as a function of the interlayer distance (*d*_0_) between ML MnSe_2_ and ML As. During the variation of *d*_0_, the MnSe_2_/As heterostructure was not re-optimized. Specifically, the structure of MnSe_2_ was maintained identical to that of the pristine MnSe_2_ ML. As illustrated in Figure 8, the MAE of the MnSe_2_/As heterostructures under different interlayer distances exhibit an oscillatory behavior. When *d*_0_ falls within the range between the vdW distance and 2.96 Å, the MnSe_2_/As heterostructures exhibit PMA. The PMA reaches a peak of approximately 2 meV at *d*_0_ = 2.56 Å. When *d*_0_ is slightly larger than 2.96 Å, a transition from PMA to IMA takes place. The IMA decreases gradually with further increasing the *d*_0_. These results establish interlayer distance as a critical knob for tailoring magnetic anisotropy in vdW systems, with implications for designing vertical strain-responsive spintronic devices.

**Figure 9:**
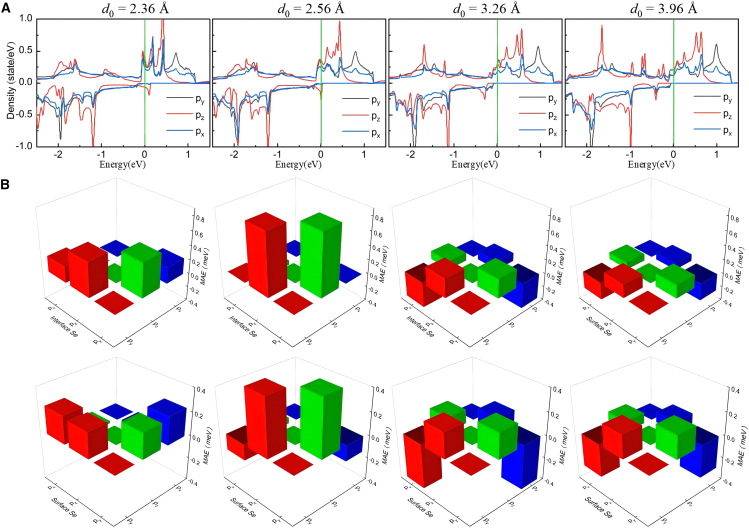
PDOS and orbital-projected MAE contribution of Se atoms in MnSe_2_/As heterostructures under different interlayer distances (A) PDOS of Se atoms. (B) Contribution to MAE from Se atoms. In (B), the upper and lower rows represent the contribution to MAE from the interfacial and surface Se atoms, respectively.

Via Bader charge analysis, it is known that there is an inverse proportional relationship between the amount of charge transfer and the interlayer distance (see the inset in Figure 8). When *d*_0_ decreases, the charge transfer at the heterointerface will be intensified, causing the built-in electric field formed between the two layers to be enhanced accordingly. Moreover, this built-in electric field will further modulate the spin-energy level splitting and ultimately affect the MAE. We have further enumerated the PDOS and the orbital-projected MAE contributions from Se atoms in the MnSe_2_/As heterostructure under different interlayer distances, as shown in Figures 9A and 9B. The results reveal that the modulation of MAE arises from variations in the orbital contributions of both interfacial and surface Se atoms. Considering the compressive strain in the vertical direction (i.e., *d*_0_ varying from 2.76 Å to 2.56 Å to 2.36 Å), the spin-down *p*_*z*_ states near the *E*_F_ continuously shift toward higher energies (rightward), transitioning from occupied states to unoccupied states (see Figure 5, Figure 9A and 5B). As detailed in Table S1 in supplemental information, the coupling of $\left(p_{x\left(y\right)}^{u^{+}},p_{y\left(x\right)}^{o^{-}}\right)$ states yields a value of −1, which contributes to IMA. The coupling of $\left(p_{z}^{u^{+}},p_{x\left(y\right)}^{o^{-}}\right)$ and $\left(p_{x\left(y\right)}^{u^{+}},p_{y\left(x\right)}^{o^{+}}\right)$ states results in a value of + 1, favoring PMA. The orbital-projected MAE contributions directly reflect PDOS variations, as depicted in Figures 9B, 4E, and 4F. From 2.76 Å to 2.56 Å to 2.36 Å, the positive SOC contribution to MAE via the (*p*_*z*_, *p*_*y*_) orbitals gradually decreases, while the SOC contribution between the (*p*_*x*_, *p*_*y*_) orbitals transitions from negative to positive. Moreover, the Se-*p* states show the smallest energy gap between occupied and unoccupied levels at *d*_0_ = 2.56 Å. Based on the perturbation theory, this condition corresponds to a maximum PMA. Under vertical tensile strain (i.e., *d*_0_ varying from 2.76 Å to 3.26 Å to 3.96 Å), the easy magnetization axis switches from out-of-plane to in-plane, mainly attributed to the variations of *p*_*z*_ states near the *E*_F_. As shown in Figures 9A and 5B, the $p_{z}^{o^{-}}$ states near the *E*_F_ shift toward lower energies (leftward), while the densities of $p_{z}^{o^{+}}$ states near the *E*_F_ increase. As a result, the positive SOC contribution to MAE via the (*p*_*z*_, *p*_*y*_) orbitals gradually decreases.

### Effect of As-layer thickness on MAE

At last, to further explore the role of dimensional effects, we have investigated the impact of the number of As layers on the MAE of the MnSe_2_/As heterostructures. As shown in Figure S5 of the supplemental information, the structures consisting of a single-layer MnSe_2_ coupled with 2–4 layers of As are optimized, with the lattice constants *a*_0_ are 3.717, 3.736, and 3.748 Å, respectively. The results show that an increasing trend in IMA is observed as the thickness of As layers increases from 2 to 4. Specifically, the MAE values for heterostructures with 2-, 3-, and 4-layer As are −0.436, −0.805, and −0.951 meV, respectively. Notably, the stacking configuration for the multi-layer As heterostructures differs from the single-layer As case, as the former was selected based on its lowest-energy configuration. Further details are provided in Figure S6 of the supplemental information. By comparing the PDOS of Se atoms in the MnSe_2_/As heterostructures with 1-layer and 2-layer As (as depicted in the Figures 5B and S7), the transition of the easy magnetization axis from out-of-plane to in-plane is mainly attributed to the $p_{z}^{o^{-}}$ states, the $p_{y\left(x\right)}^{o^{-}}$ states near the *E*_F_ moving toward lower energies (rightward), and the $p_{z}^{o^{-}}$ states increase. When the number of As layers increases from 2 to 4, IMA increases by 100%. This is primarily because the $p_{z}^{o^{-}}$ states near the *E*_F_ decrease, while the $p_{z}^{o^{+}}$ states increase. Combined with second-order perturbation theory, the variation trend of the MAE is explained.

### Conclusions

This study reveals the tunability of magnetic anisotropy in the MnSe_2_/As vdW heterostructures through first-principles calculations. The pure MnSe_2_ ML exhibits weak IMA, whereas the heterostructure demonstrates significantly enhanced PMA. This transition is driven by interlayer charge transfer, which induces a built-in electric field that modulates the crystal field and orbital coupling of Se atoms. The PMA of the MnSe_2_/As heterostructures can be flexibly tuned by external electric fields. A negative field enhances charge transfer, leading to strengthened PMA, while a positive field weakens it. The interlayer distance plays a critical role in determining the anisotropy. MAE exhibits oscillatory behavior between in-plane and out-of-plane orientations, with PMA peaking at ∼2 meV/cell when the distance is 2.56 Å and transitioning to IMA beyond 2.96 Å. Increasing the thickness of As layers (2–4 layers) enhances IMA, primarily due to changes in the occupation of Se *p*_*z*_ orbitals. These results may offer valuable guidance for the preparation of vdW heterostructures and related devices.

### Limitations of the study

First, the calculations are based on idealized structural models without considering possible defects, impurities, or interfacial disorder that may occur in experimentally synthesized samples. Second, the study focuses on specific stacking configurations, while real-world heterostructures may exhibit more complex stacking sequences or variations in interfacial alignment. Third, the external electric fields and interlayer distance variations applied in the simulations may not be fully achievable or controllable in experimental settings.

## Resource availability

### Lead contact

Requests for further information and resources should be directed to and will be fulfilled by the lead contact, Wei Chen (po_ze_xi@126.com).

### Materials availability

This study did not generate new unique reagents.

### Data and code availability

Data reported in this paper will be shared by the lead contact upon request. There is no code associated with this work. Any additional information required to reanalyze the data reported in this paper is available from the lead contact upon request.

## Acknowledgments

This work was supported by the 10.13039/501100004735Hunan Provincial Natural Science Foundation of China (2023JJ40074 and 2025JJ50382). The Program of Changsha Excellent Young Talents (kq1905005, kq2009076, kq2106071, kq2206052, and kq2305029).

## Author contributions

Conceptualization, W.C. and J.L.; investigation, W.C. and Y.L.; writing – original draft preparation, W.C.; writing – review and editing, J.L. and Y.G.

## Declaration of interests

The authors declare no competing financial interest.

## Declaration of generative AI and AI-assisted technologies in the writing process

During the preparation of this work, the authors used Deepseek in order to assist with formatting and refine technical language. After using this tool, the authors reviewed and edited the content as needed and take full responsibility for the content of the publication.

## STAR★Methods

### Key resources table

**Table:** 

REAGENT or RESOURCE	SOURCE	IDENTIFIER
**Software and algorithms**		

VASP	Kresse and Furthmü ller	https://www.vasp.at/
OriginPro 2025	OriginLab Corporation	https://www.originlab.com/
Wannier90	Marzari, Vanderbilt, Sancho et al.	https://wannier.org/
TB2J	Python package	https://tb2j.readthedocs.io/en/latest/

### Experimental model and study participant details

This study does not use experimental methods typical in the life sciences.

### Method details

The detailed numerical setting in first-principles calculations have been presented in computational details.

### Quantification and statistical analysis

Our study does not include statistical analysis or quantification.

### Additional resources

There are no additional resources needed to be declared in this manuscript, additional requests for this can be made by contacting the lead contact.
