# Supplementary material for: Spin reorientation and controllable magnetic anisotropy in two-dimensional MnSe2/As van der Waals heterostructures
Source: iScience. 2025 Oct 23;28(11):113830. doi: 10.1016/j.isci.2025.113830 (PMC12637063; doi:10.1016/j.isci.2025.113830)
Supplement: Document S1. S1–S7, Tables S1 and Data S1 [file mmc1.pdf]

iScience, Volume *28*

Supplemental information

Spin reorientation and controllable magnetic anisotropy in two-dimensional MnSe2/As van der Waals heterostructures

Wei Chen, Yunpeng Lan, Jujian Liao, and Youneng Guo

Supplemental information

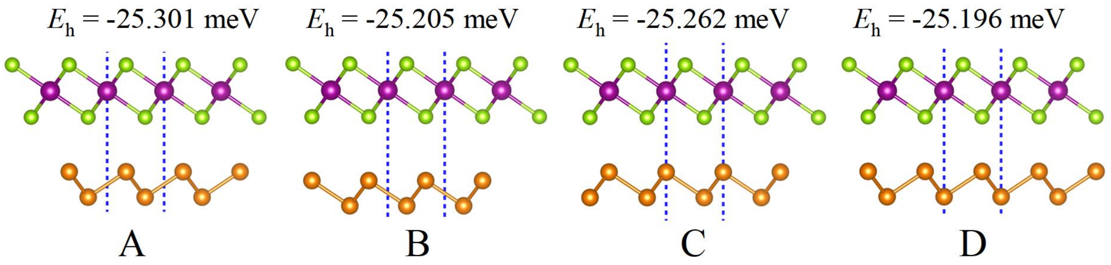

Figure S1. Side views of four different stacking configurations of $MnSe_2$/As heterostructures. (A-D) The energies (E_h) of configurations (B), (C), and (D) are all higher than that of (A). Therefore, configuration (A) is the most stable.

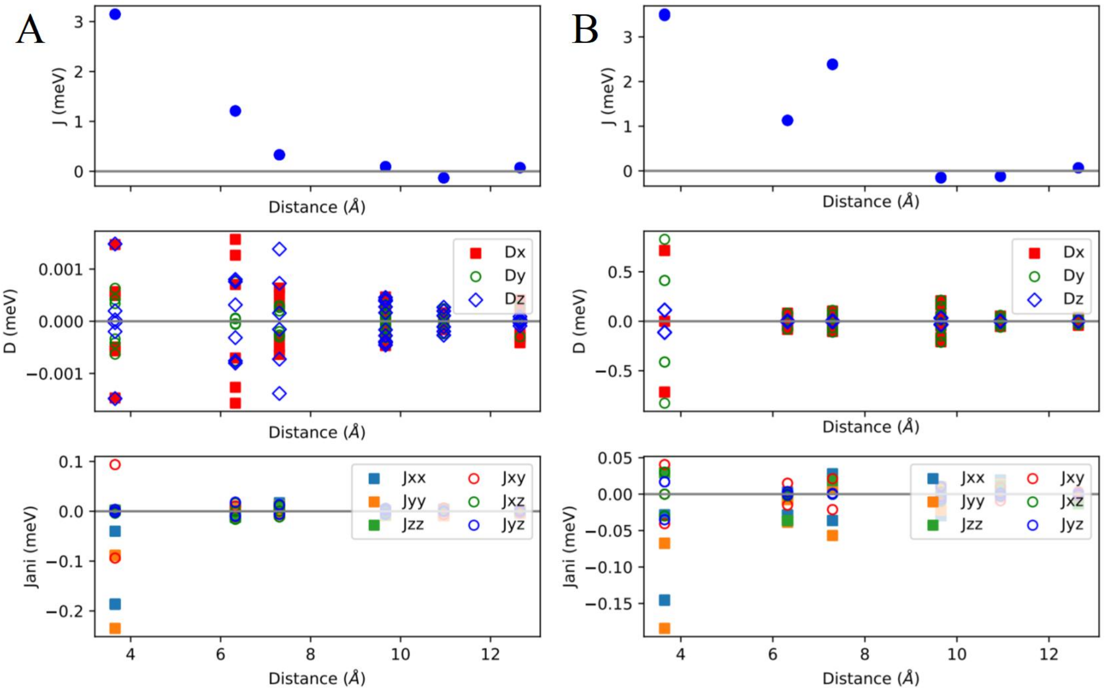

Figure S2. Exchange constants for two systems. (A) Pure $MnSe_2$ monolayer. (B) $MnSe_2$/As heterostructure. In (A) and (B), *J*, *D* and *Jani* represent the isotropic, symmetric anisotropic, and antisymmetric anisotropic exchange constants, respectively. There are multiple pairs of exchange constants at the same distance, resulting in multiple overlapping points at each position.

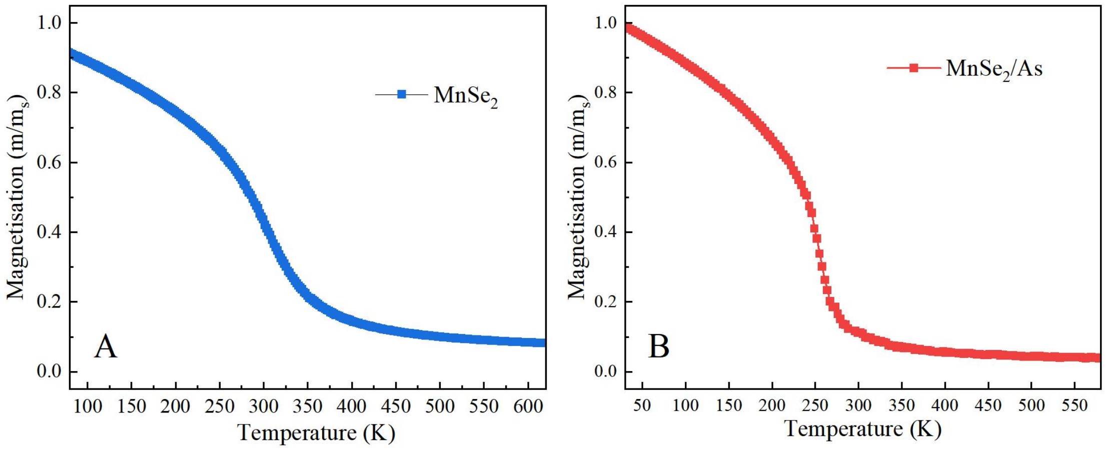

Figure S3. Variation of the average magnetic moment with temperature in two systems. (A) Pure $MnSe_2$ monolayer. (B) $MnSe_2$/As heterostructure.

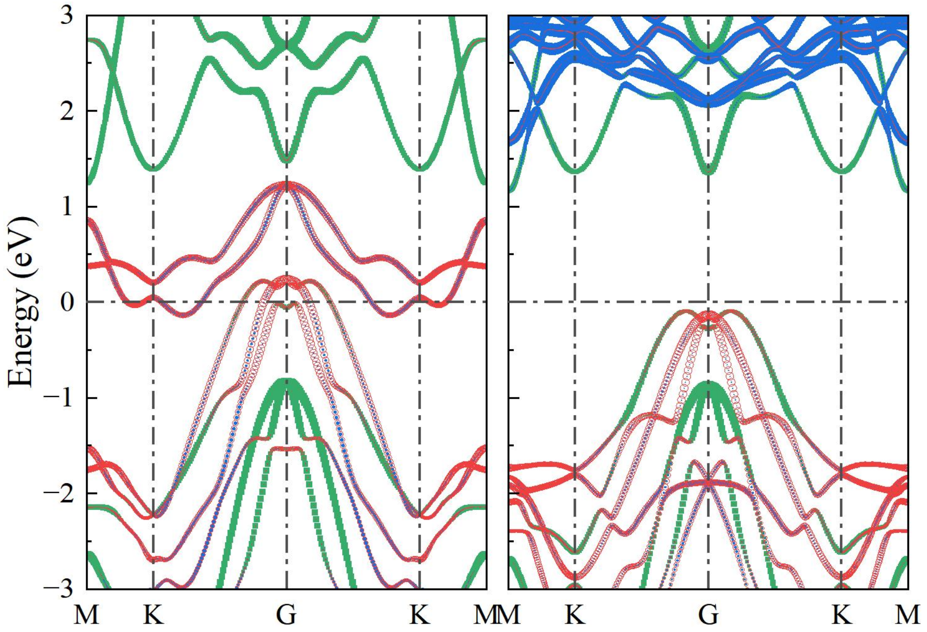

Figure S4. Element-projected band structures of $MnSe_2$/As heterostructure. Green, blue, and red represent the contributions from As, Mn, and Se atoms, respectively.

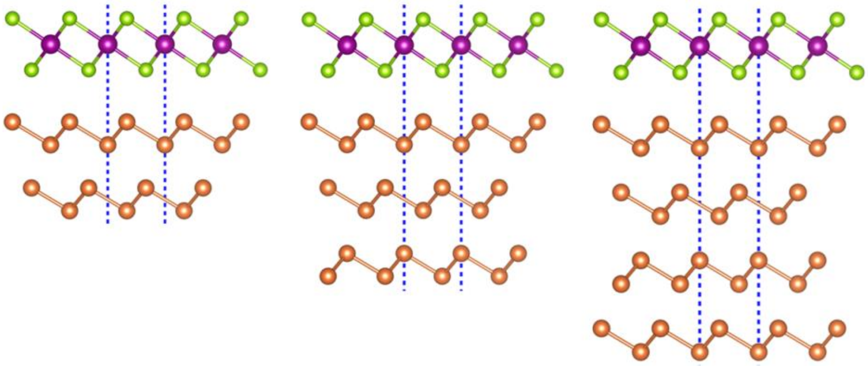

Figure S5. $MnSe_2$/As heterostructures consists of a single-layer $MnSe_2$ coupled with 2 ~ 4 layers of As.

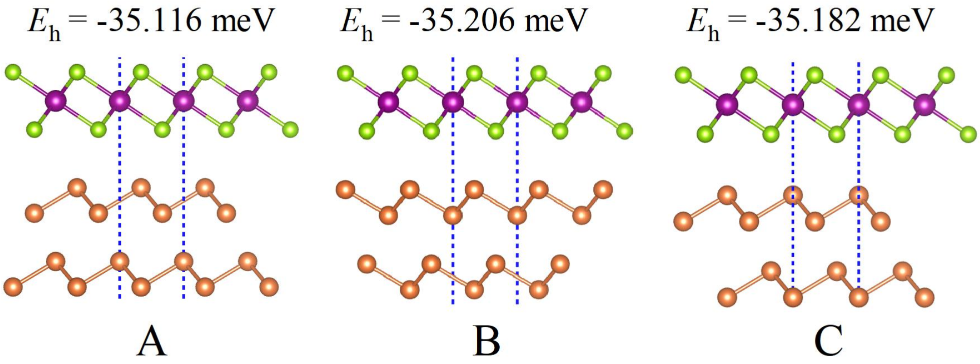

Figure S6. Side views of three different stacking configurations of the $MnSe_2$/(2-layer)As heterostructures. (A-C) The energies (E_h) of configurations (A) and (C) are higher than that of (B). Therefore, configuration (B) is the most stable.

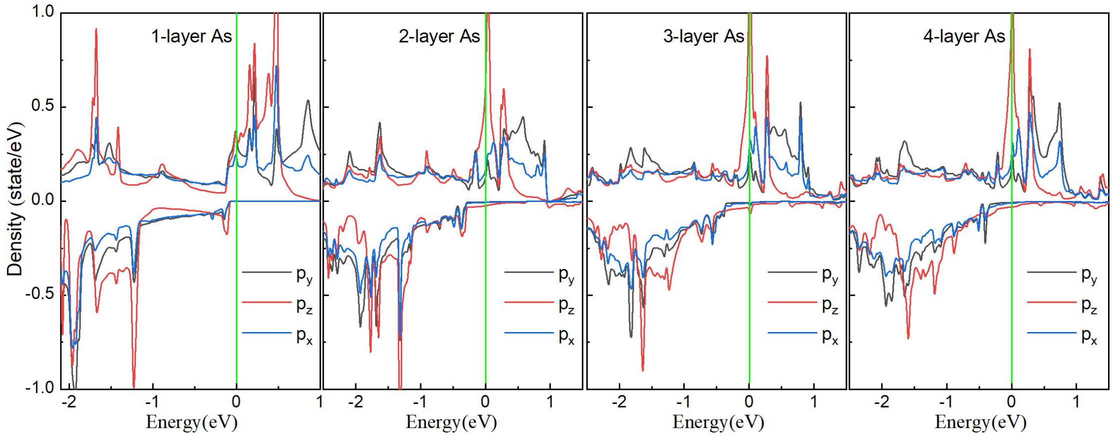

Figure S7. PDOS of Se atoms for $MnSe_2$/As heterostructures consisting of a single-layer $MnSe_2$ coupled with 1 ~ 4 layers of As.

Table S1. SOC matrix differences for Se-p orbitals between magnetization along *x*-axis [100] and z-axis [001] in equations (1). u^-, o^+, and o^- represent the unoccupied spin-down states, occupied spin-up and spin-down states, respectively.

	o^+			o^-		
u^-	p_y	p_z	p_x	p_y	p_z	p_x
p_y	0	1	-1	0	-1	1
p_z	1	0	0	-1	0	0
p_x	-1	0	0	1	0	0

Data S1. Exchange interaction and Curie temperature

To more accurately describe the exchange interactions, we consider a Heisenberg model with multiple exchange anisotropies:

$$\mathcal{H}_{\text{exch}} = -\frac{1}{2}\sum_{i\neq j} \mathrm{S}_i^{\alpha}\mathcal{J}_{ij}^{\alpha\beta}\mathrm{S}_j^{\beta} - \sum_i D(S_i^z)^2, \quad \alpha,\beta = x,y,z$$

Where S_i and S_j are spin vectors located at lattice sites i and j, D is the uniaxial anisotropy, and $\mathcal{J}_{ij}$ is the exchange tensor expressed as $\mathcal{J}_{ij} = J_{ij}\mathbf{I} + \mathcal{J}_{ij}^S + \mathcal{J}_{ij}^A$, which can be decomposed into isotropic exchange J_{ij} ($\mathbf{I}$ is the unit tensor), symmetric anisotropic exchange $\mathcal{J}_{ij}^S$, and antisymmetric anisotropic exchange $\mathcal{J}_{ij}^A$ corresponding to the DMI. We initiate the process with three converged DFT + SOC + U calculations, corresponding to magnetic moments aligned along the x, y, and z axes, respectively, and considering all exchange interactions within an 13 Å distance using the TB2J method. In fact, all ij pairs are generated for the spin labeled i in the center cell of a 18 × 18 × 1 supercell, while the exchange interaction becomes negligible beyond a distance of 13 Å. TB2J treats rigid spin rotation as perturbation on the electronic structure using the magnetic force theorem. Energy variations are calculated from the Green's function of the tight-binding Hamiltonian for Wannier functions constructed using DFT+SOC+U.
